# *Cyfip1* Haploinsufficiency Does Not Alter GABA_A_ Receptor δ-Subunit Expression and Tonic Inhibition in Dentate Gyrus PV^+^ Interneurons and Granule Cells

**DOI:** 10.1523/ENEURO.0364-18.2019

**Published:** 2019-06-28

**Authors:** Simon Trent, Jeremy Hall, William M. Connelly, Adam C. Errington

**Affiliations:** 1 Neuroscience and Mental Health Research Institute, School of Medicine, Cardiff University, Cardiff, CF24 4HQ, United Kingdom; 2 School of Medicine, University of Tasmania, Hobart, Tasmania 7000, Australia

**Keywords:** CYFIP1, dentate gyrus, GABA, neurodevelopmental, tonic inhibition

## Abstract

Copy number variation (CNV) at chromosomal region 15q11.2 is linked to increased risk of neurodevelopmental disorders including autism and schizophrenia. A significant gene at this locus is cytoplasmic fragile X mental retardation protein (FMRP) interacting protein 1 (*CYFIP1*). CYFIP1 protein interacts with FMRP, whose monogenic absence causes fragile X syndrome (FXS). *Fmrp* knock-out has been shown to reduce tonic GABAergic inhibition by interacting with the δ-subunit of the GABA_A_ receptor (GABA_A_R). Using *in situ* hybridization (ISH), qPCR, Western blotting techniques, and patch clamp electrophysiology in brain slices from a *Cyfip1* haploinsufficient mouse, we examined δ-subunit mediated tonic inhibition in the dentate gyrus (DG). In wild-type (WT) mice, DG granule cells (DGGCs) responded to the δ-subunit-selective agonist THIP with significantly increased tonic currents. In heterozygous mice, no significant difference was observed in THIP-evoked currents in DGGCs. Phasic GABAergic inhibition in DGGC was also unaltered with no difference in properties of spontaneous IPSCs (sIPSCs). Additionally, we demonstrate that DG granule cell layer (GCL) parvalbumin-positive interneurons (PV^+^-INs) have functional δ-subunit-mediated tonic GABAergic currents which, unlike DGGC, are also modulated by the α_1_-selective drug zolpidem. Similar to DGGC, both IPSCs and THIP-evoked currents in PV^+^-INs were not different between *Cyfip1* heterozygous and WT mice. Supporting our electrophysiological data, we found no significant change in hippocampal δ-subunit mRNA expression or protein level and no change in α_1_/α_4_-subunit mRNA expression. Thus, *Cyfip1* haploinsufficiency, mimicking human 15q11.2 microdeletion syndrome, does not alter hippocampal phasic or tonic GABAergic inhibition, substantially differing from the *Fmrp* knock-out mouse model.

## Significance Statement

*CYFIP1* is a candidate risk gene for neurodevelopmental and neuropsychiatric disorders. CYFIP1 protein interacts with FMRP whose loss downregulates tonic GABAergic inhibition via interaction with the δ-subunit of the GABA_A_ receptor (GABA_A_R). Here, however, we report that reduced *Cyfip1* dosage in mice does not alter tonic GABAergic inhibition in granule cells and parvalbumin-positive interneurons (PV^+^-INs) of the dentate gyrus (DG), a region rich in δ-subunit expression. Despite these negative findings, our data does demonstrate that PV^+^-INs of the DG granule cell layer (GCL) are functionally regulated by tonic GABAergic inhibition, and in contrast to granule cells, this involves receptors incorporating both δ- and α_1_-subunits. Thus, GCL excitatory neurons and PV^+^-INs may be differentially modulated by subunit-selective GABA receptor targeting drugs.

## Introduction

Cytoplasmic fragile X mental retardation protein (FMRP) interacting protein 1 (*CYFIP1*) is a gene found in the 15q11.2 locus of the human genome (Chai et al., 2003). Copy number variations (CNVs) at this region, including both microdeletions and microduplications, span *CYFIP1* and three other genes (*NIPA1*, *NIPA2*, *TUBGCP5*) and have been strongly linked by genomic studies to increased risk of developing neuropsychiatric and neurodevelopmental disorders including autism spectrum disorder and schizophrenia (Stefansson et al., 2008; Burnside et al., 2011; Kirov et al., 2012). *CYFIP1* has a number of known functions and the protein it encodes (CYFIP1) interacts with several key signaling complexes. For example, CYFIP1 is involved in the maturation and maintenance of dendritic complexity and dendritic spines by suppressing the WAVE regulatory complex and regulating actin cytoskeletal dynamics (De Rubeis et al., 2013; Pathania et al., 2014). Rodent models of Cyfip1 haploinsufficiency, broadly modeling reduced gene dosage of *Cyfip1* in 15q11.2 CNV carriers, reveal behavioral deficits in the form of altered extinction in inhibitory avoidance, although wider effects on anxiety and learning were not observed (Bozdagi et al., 2012).

As indicated by its name, CYFIP1 is an important functional partner of the RNA-binding protein FMRP (Schenck et al., 2001; Napoli et al., 2008), which regulates dendritic targeting of mRNAs (Bassell and Warren, 2008), influences mRNA stability (De Rubeis and Bagni, 2010) and represses protein translation of ∼800 neuronal mRNA “FMRP targets” (Darnell et al., 2001; Hou et al., 2006). The transcriptional silencing of the FMRP gene *FMR1* causes fragile X syndrome (FXS), which is characterized by a range of physical, behavioral and cognitive deficits (Garber et al., 2008) and is the leading monogenic cause of autism and intellectual disability (Santoro et al., 2012).

In comparison to CYFIP1, the molecular pathways disrupted by FMRP loss have been more extensively characterized. One significant effect of FMRP loss is disruption of GABAergic signaling across brain regions including the hippocampus, cortex, and amygdala (Paluszkiewicz et al., 2011; Braat et al., 2015). FMRP is expressed in GABAergic interneurons throughout development suggesting an important role in interneuron maturation and function (Feng et al., 1997) and a subset of GABAergic signaling mRNAs appear to be under the regulation of FMRP (El Idrissi et al., 2005; Darnell et al., 2011). *Fmr1* KO animal studies have revealed that Fmrp loss produces significant pre- and postsynaptic effects on GABAergic signaling. Changes in the level of the GABA synthesizing enzyme glutamatic acid decarboxylase (GAD65/67), the GABA transporter 1 (GAT-1) and enzymes responsible for GABA breakdown (GABA-T and SSADH) have all been associated with loss of FMRP (Martin and Huntsman, 2014). Postsynaptically, decreased mRNA expression and/or protein levels for at least eight GABA_A_ receptor (GABA_A_R) subunits (α_1_, α_3_, α_4_, β_1_, β_2_, γ_1_, γ_2_, and δ) have been described in amygdala, cortex, and hippocampus (Braat et al., 2015; Sabanov et al., 2017; Zhang et al., 2017).

In particular, deficits in tonic GABAergic inhibition have been implicated. FMRP has been shown to bind to the GABA_A_R δ-subunit (Miyashiro et al., 2003; Dictenberg et al., 2008) and *Fmrp* knock-out reduces δ-subunit mRNA and protein expression in the amygdala, cerebellum, cortex, and dentate gyrus (DG) of a mouse model of FXS (D’Hulst et al., 2006; Curia et al., 2009; Braat et al., 2015; Zhang et al., 2017). Moreover, the GABA_A_R δ-subunit-selective agonist THIP and the neurosteroid ganaxolone ameliorate symptoms in a mouse model of FXS (Olmos-Serrano et al., 2011; Braat et al., 2015). Importantly, the δ-subunit is exclusively found in extrasynaptic GABA_A_Rs (eGABA_A_Rs) and mediates tonic inhibition across brain regions, although in the hippocampus it is almost exclusively expressed in the DG (Pirker et al., 2000; Zheng et al., 2009; Milenkovic et al., 2013). Thus, the current view is that disrupted tonic GABAergic inhibition may be a major contributing in FXS and that modulation of GABAergic signaling is a potential route for therapeutic intervention in this disorder (Braat et al., 2015).

Therefore, due to the association of *CYFIP1* with neuropsychiatric disorders, its known interaction with FMRP and the effects of FMRP on GABA signaling, we have used a *Cyfip1* haploinsufficient mouse to explore the effects of Cyfip1 on GABAergic inhibition. We find that, unlike Fmrp loss, *Cyfip1* haploinsufficiency does not reduce GABA_A_R δ-subunit expression in the hippocampus. Electrophysiological experiments show that neither phasic IPSCs nor tonic GABAergic inhibition is changed in DG granule cells (DGGCs) or granule cell layer (GCL) parvalbumin-positive interneurons (PV^+^-INs). Thus, in mouse hippocampus, haploinsufficiency of *Cyfip1* does not disrupt GABAergic signaling in a similar manner to *Fmrp* knock-out. Nonetheless, our findings reveal that GCL PV^+^-INs do, as previously suggested (Milenkovic et al., 2013), express functional eGABA_A_Rs containing δ-subunits that contribute to tonic inhibition in these cells. Unlike DGGC (Nusser and Mody, 2002), a fraction of the tonic current in GCL PV^+^-INs is modulated by the α_1_-selective ligand zolpidem suggesting these cells may express α_1_-subunit-containing eGABA_A_Rs.

## Materials and Methods

### Animals

Experiments involving recordings from DGGCs were performed on *Cyfip1* heterozygous (*Cyfip1^+/–^*) and wild-type (WT) mice. The *Cyfip1* mouse line (Allele: *Cyfip1^tm2a(EUCOMM)Wtsi^* ^)^ was generated using the “knockout-first” strategy by the Wellcome Trust Sanger Institute as part of the International Knockout Mouse Consortium (IKMC) on the C57BL/6N Taconic background. We obtained pairs of breeding mice (B6NTac;B6N-A^tm1Brd^ Cyfip1^tm2a(EUCOMM)Wtsi/^WtsiH) from the EMMA mouse repository (Infrafrontier Mouse Disease Models, RRID: IMSR_EM:06868). Experimental animals were generated by crossing male *Cyfip1^+/–^* mice with WT female C57BL/6J mice (RRID: IMSR_JAX:000664), generating hybrid C57BL/6J/6N Cyfip1^+/–^ and WT littermates. Animals were genotyped following the procedure recommended by the Sanger Institute. For experiments involving recordings from PV^+^-INs in the DG GCL, we crossed a PV-Cre knock-in mouse (B6;129P2-Pvalb^tm1(cre)Arbr^/J, RRID: IMSR_JAX:008069) with a Cre-dependent tdTomato reporter mouse (B6.Cg-Gt(ROSA)26Sor^tm14(CAG-tdTomato)Hze^/J, RRID: IMSR_JAX:007914) to drive expression of the red fluorescent protein tdTomato in PV^+^ cells. These animals were then subsequently crossed with Cyfip1^+/–^ mice to produce PV^+^TdCyfip1^WT^/^+/–^ mice. Electrophysiological experiments and protein and mRNA expression experiments were performed on adolescent (five- to seven-week-old) or adult (3.5- to six-month-old) mice. All protein and mRNA expression work was conducted on behaviorally naive, adult male 6J/6N hybrid mice and further molecular work was performed on behaviorally naive adolescent male 6J/6N mice. All animal procedures were performed in accordance with Cardiff University's animal care committee's regulations and the Home Office Animals (Scientific Procedures) Act, 1986 UK.

### *In situ* hybridization (ISH), qPCR, and Western blot analysis

For all molecular work, mice were sacrificed by carbon dioxide inhalation and whole brains were extracted. For *in situ* hybridization (ISH), whole brains were snap-frozen and stored at –80°C, while for quantitative real-time PCR (qRT-PCR) and protein immunoblotting by Western blotting, the whole hippocampus from both brain hemispheres were dissected free-hand and frozen on dry ice before storage at –80°C.

### ISH

Coronal brain sections (14 μm) were cut using a cryostat (–20°C, Leica Microsystems) and mounted on poly-L-lysine coated glass slides. Each slide consisted of one slice from six separate brains, matched for hippocampal region (Paxinos and Franklin, 2004) and counterbalanced across genotype. Two series of the dorsal hippocampus were produced representing all experimental animals (*n* = 6 per genotype). Slides were fixed in 4% PFA solution, followed by dehydration in ethanol and stored in 95% EtOH at 4°C until required. Slides were processed in parallel as an internal technical control.

The protocol for semi-quantitative ISH using a 3’-end radiolabeled oligonucleotide (45 mer) with [ɑ-35S]dATP was broadly similar to previous work (Kirtley and Thomas, 2010; Clifton et al., 2017). Briefly, an oligonucleotide probe for mouse Gabrd was bioinformatically designed (NM_008072.2, FASTA, NCBI) to contain 45 base pairs, a 50% AT:CG ratio, contain no more than three consecutive matching bases and detect all known *Gabrd* mouse transcripts (NCBI and Ensembl). The *Gabrd* probe sequence (3’–5’) was: TCCAT GTCAC AGGCC ACTGT GGAGG TGATG CGGAT GCTGT ATAAA, binding to the complementary murine *Gabrd* nucleotide sequence (nucleotide position 607–563).

The *Gabrd* probe was commercially synthesized (Sigma-Aldrich) and diluted in phosphate buffer (1 μg/μl, pH 7) and further diluted to a working concentration of 5 ng/μl (in sterile water). *Gabrd* oligonucleotide probe (1 μl) was added to 6.5 μl deionized water, 2.5 μl terminal deoxynucleotidyl transferase buffer (Promega), 1 μl terminal deoxynucleotidyl transferase (Promega), and 1 μl deoxyadenosine 5’-(α-thio)triphosphate [35S] (dATP; PerkinElmer), before being incubated at 30°C for 1 h for 3’-end nucleotide labeling. After incubation, the labeled nucleotide went through clean-up via Qiaquick Nucleotide Removal kits (QIAGEN, as per manufacturer’s protocol) and 2 μl dithiothreitol (DTT; 1 M) was added to the eluted oligonucleotide. Activity of labeled *Gabrd* probe was measured (HIDEX Triathler liquid scintillation counter) and within a range of 60,000–250,000 CPM/µl.

Three consecutive slides of brain sections (per series) were selected for ISH, with two slides used to define total specific (TS) hybridization levels (i.e., technical repeat included) and 1 slide to define the non-specific (NS) hybridization signal. Radiolabeled probes were applied at a level of 200,000 cpm per slide. A master mix for the *Gabrd* probe was made including radiolabeled probe, 2 μl of DTT (1 M) and 100-μl hybridization buffer (HYB) per slide (for further details on HYB, see Wisden and Morris, 1994). This master mix solution was applied to two TS slides per series (100 μl/slide), and then unlabeled probe was added to the remaining master mix (8:1 ratio of unlabeled/labeled probe) and this was subsequently added to NS allocated slides (100 μl). Parafilm strips were used to form the necessary matrix for ISH to occur, while all slides were sealed in humidified plastic chambers and incubated at 42°C overnight. Parafilm coverslips were subsequently removed in 1 × SSC at room temperature (RT). Slides were then washed in 1 × SSC at 52°C for 1 h, rinsed in 0.1 × SSC, and dehydrated in ethanol.

As per Kirtley and Thomas (2010), autoradiographs were generated using radiographic film exposed to a quantitative C14 ladder and TS/NS slides for 7 d and developed. Autoradiograph densitometry was performed (ImageJ) blind to genotype, whereby NS values were subtracted from TS values to provide a Specific activity value, providing a measure of mRNA expression.

### qRT-PCR

RNA was extracted from dissected whole hippocampus using RNeasy kits (QIAGEN), followed by DNase treatment of RNA (TURBO DNA-free kit, Thermo Fisher Scientific), and converted to cDNA (RNA to cDNA EcoDry Premix, Random Hexamers, Clontech, Takara). cDNA samples were prepared in triplicate in 96-well reaction plates for SYBR-green-based qRT-PCR (SensiFAST, HI-ROX, Bioline), according to manufacturer’s instructions, using a StepOnePlus System (Applied Biosystems, Thermo Fisher Scientific). *Gabrd*-specific primers, alongside *Gapdh* and *Hprt* primers (housekeeping genes), were bioinformatically designed to span at least one exon-exon boundary and to match only for its target mRNA sequence in mouse (primer-BLAST and nBLAST, NCBI), before being commercially synthesized (Sigma-Aldrich). Primer efficiencies were experimentally determined through a dilution series of experimentally separate WT mouse hippocampal cDNA (efficiency of 90–110% was required, annealing temperature set at 60°C). All samples were run in triplicate and individual ΔCt values (relative to *Gapdh* and *Hprt*) were used to quantify mRNA gene expression. Primers used for qRT-PCR were as follows: *Gabrd*, forward GGGCAGAGATGGATGTGAGG and reverse CTTGACGACGGGAGATAGCC (targeting exon 8–9); *Gapdh*, forward GAACATCATCCCTGCATCCA and reverse CCAGTGAGCTTCCCGTTCA; and *Hprt*, forward TTGCTCGAGATGTCATGAAGGA and reverse AATGTAATCCAGCAGGTCAGCAA.

### Immunoblotting by Western blotting

Hippocampal tissue was homogenized manually with a glass Dounce homogenizer in 1 ml of ice-cold lysis RIPA buffer (Thermo Fisher Scientific) containing protease inhibitors (cOmplete Mini EDTA-free Protease Inhibitor, Roche, Sigma-Aldrich, 1 tablet/10 ml RIPA). The homogenates were centrifuged at 12,000 rpm for 20 min at 4°C and aliquots of supernatant containing proteins stored at –80°C. Total protein was quantified using Pierce BCA Protein kit Assay, as per manufacturer’s instructions (Thermo Fisher Scientific) and a 1:1 ratio of 40 µg of protein sample was added to 2 × Laemmli sample buffer (containing 1:20 β-mercaptoethanol, Bio-Rad). Samples were denatured at 95°C for 5 min, loaded alternatively by genotype and separated on a 4–12% gradient Bis-Tris Midi gel (NuPAGE, Thermo Fisher Scientific) in 1× Bolt MES SDS Running buffer (Thermo Fisher Scientific) at a constant voltage of 115 V for 1 h. Transfer was performed in 1× Bolt Transfer buffer (Thermo Fisher Scientific) to GE Healthcare Protran nitrocellulose membranes (GE Healthcare Life Sciences) at a constant voltage of 85 V for 2 h 15 min at 4°C.

Blots were blocked in 5% non-fat milk (GE Healthcare ECL Blocking Agent, GE Healthcare Life Sciences) in 0.1 M Tris-buffered saline solution containing 0.2% Tween 20 (TBST), and this TBST solution was used for all subsequent washes. Primary and fluorescent secondary antibodies were similarly diluted in TBST containing 0.2% Tween 20 and 5% milk and they were used at the following concentrations: GABA-A R δ (Novus Biologicals, RRID: AB_2107256), 1:500; calnexin (Abcam, RRID: AB_2069006), 1:5000; and IRDye 680RD goat anti-rabbit IgG (Li-Cor, RRID: AB_10956166), 1:15,000. Incubation of blots in primary antibody solutions were performed at 4°C overnight, while fluorescent secondary antibodies were for 1 h at RT. Blots were visualized using the 700-nm channel of the Odyssey CLx Imaging System (Li-Cor) and densitometric quantification was performed on scanned blot films using ImageStudio Lite software (Li-Cor). The raw fluorescent signal of each GABA-A R δ band per sample was divided by its own protein loading control, calnexin (with background subtraction). The WTs on the entire blot were then averaged together and normalized to 100%, while across-blot variance was minimized by giving each individual Cyfip1^+/–^ signal relative to the averaged total of all WT lanes.

### Brain slice preparation and electrophysiology

Animals of either sex were deeply anaesthetized using isoflurane, decapitated and their brains removed into chilled (1–3°C) cutting solution containing 60 mM sucrose, 85 mM Nacl, 2.5 mM KCl, 1 mM CaCl_2_, 2 mM MgCl_2_, 1.25 mM NaH_2_PO_4_, 25 mM NaHCO_3_, 25 mM D-glucose, 3 mM kynurenic acid, and 0.045 mM indomethacin. Horizontal hippocampal brain slices (300 μm) containing the DG were prepared from adolescent and adult WT and heterozygous *Cyfip1* and PV^+^TdCyfip1 mice, stored for 20 min at 35°C in sucrose-containing solution and subsequently maintained at RT in artificial CSF (aCSF) containing 125 mM NaCl, 2.5 mM KCl, 1 mM CaCl_2_, 1 mM MgCl_2_, 1.25 mM NaH_2_PO_4_, 25 mM NaHCO_3_, and 25 mM D-glucose (305 mOsm) then used within 4–6 h. For recording, slices were transferred to a submersion chamber continuously perfused with warmed (33–34°C) aCSF containing 125 mM NaCl, 2.5 mM KCl, 2 mM CaCl_2_, 1 mM MgCl_2_, 1.25 mM NaH_2_PO_4_, 25 mM NaHCO_3_, 25 mM D-glucose, and 3 mM kynurenic acid (305–310 mOsm, pH 7.4) at a flow rate of 3 ml min^−1^. Electrophysiological recordings were performed on DGGCs and PV^+^-INs of the DG GCL. DGGCs were identified using Dodt-contrast video microscopy and PV^+^-INs were identified by their expression of the red fluorescent protein tdTomato following two-photon excitation at λ = 900 nm (Prairie Ultima two-photon microscope, Bruker). Whole-cell voltage clamp recordings were made using a Multiclamp 700B (Molecular Devices) patch clamp amplifier with patch pipettes with resistances between 3 and 6 MΩ when filled with an internal recording solution containing 130 mM CsCl, 2 mM MgCl2, 4 mM Mg-ATP, 0.3 mM Na-GTP, 10 mM HEPES, and 0.1 mM EGTA (295 mOsm, pH 7.3) supplemented with Alexa Fluor 594 (DGGC, 50 μM, Life Technologies) or Alexa Fluor 488 (PV^+^-IN, 100 μM, Life Technologies). All experiments were performed at a holding potential (V_h_) of –70 mV unless specifically indicated elsewhere. Series resistance (R_S_) was compensated by 80% and cells showing changes of R_S_ >20% over the course of the experiment were rejected. Data were sampled at 20 kHz and low-pass filtered at 6 kHz. 4,5,6,7-tetrahydroisoxazolopyridin-3-ol (THIP, Gaboxadol), 1,2,5,6-tetrahydro-1-[2-[[(diphenylmethylene)amino]oxy]ethyl]-3-pyridinecarboxylic acid hydrochloride (NNC711, NO711), *N*,*N*,6-trimethyl-2-(4-methylphenyl)imidazo[1,2-*a*]pyridine-3-acetamide (Zolpidem), and picrotoxin (PTX) were obtained from Tocris Bio-techne.

### Data analysis and statistics

**Figure 1.**
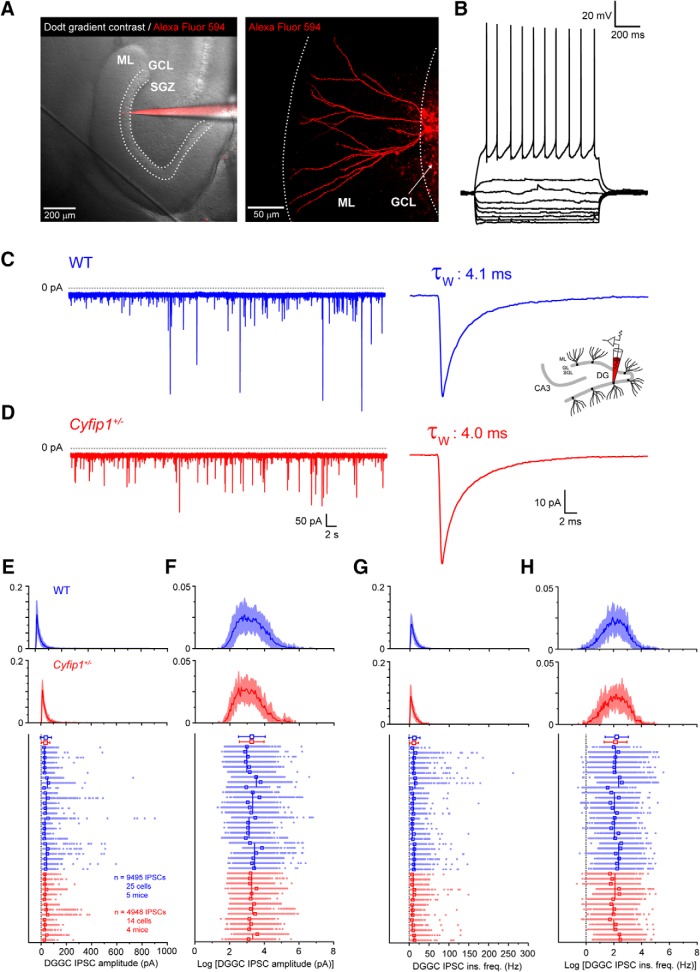
IPSCs in DGGCs of WT and *Cyfip1^+/–^* mice. ***A***, Dodt gradient contrast image of a horizontal section of the hippocampus. Dashed lines illustrate the border between the ML, GCL, and subgranular zone (SGZ). Patch pipette filled with Alexa Fluor 594 (left) illustrates the position of a mature DGGC (right), imaged by two-photon microscopy, with dendrites projecting to the edge of the ML. ***B***, Traces depicting typical electrophysiological properties of a mature DGGC. ***C***, A typical voltage clamp recording showing IPSCs from DGGC of WT mouse and the averaged IPSC from this cell. Inset schematic shown here and elsewhere signifies data recorded DGGC. ***D***, A typical voltage clamp recording showing IPSCs from DGGC of *Cyfip1^+/–^* mouse and the mean IPSC from this cell. ***E***, Histograms showing the mean (red/blue lines) and standard deviation (light blue/red shading) of the log–normal distribution of all IPSC amplitudes recorded in WT and *Cyfip1^+/–^* DGGC. Scatter plot shows the amplitude of individual IPSCs grouped by cell, mouse, and genotype. Individual IPSCs from each cell are shown as aligned dots (light blue/red) with the mean IPSC amplitude for each cell shown as an unfilled superimposed square (red/blue). Varying length vertical lines (blue/red) represent the mean IPSC amplitude for each animal with the length of the bar illustrating the number of recorded neurons from each mouse. Red/blue symbols (top) are the arithmetic mean and standard deviation of the complete dataset for each genotype. ***F***, As in ***E*** but for log-transformed IPSC amplitude data. ***G***, As in ***E*** but for IPSC instantaneous frequency data. ***H***, As in ***E*** but for log-transformed IPSC frequency data.

Spontaneous IPSCs (sIPSCs) were detected using a template search routine in pClamp 10 (Clampex, Molecular Devices) and their amplitude, frequency and integral were measured. IPSCs were automatically detected from a 20- to 60-s control baseline period and manually inspected *post hoc* for false event detection. For analysis of event frequency, the simple mean arithmetic IPSC frequency for each cell was calculated as the number of IPSCs detected divided by the length of the sampling period. The instantaneous frequency was calculated as 1/inter-IPSC interval of all recorded IPSCs. Occasional unclamped action currents were detected and these were rejected from analysis. For analysis of IPSC decay, events whose decay phase were contaminated by other IPSCs were rejected and remaining events were averaged to produce a single averaged IPSC for each cell. Each averaged IPSC was fitted with a double exponential function with two amplitude components (A and B) and two decay time constants (τ_1_ and τ_2_) to calculate the weighted decay time constant (τ_W_) by τ_W_ = [(A/A + B) τ_1_] + [(B/B + A) τ_2_]. The mean charge carried by individual IPSCs (in pC) for each cell was the integral of the averaged IPSC calculated by multiplying the amplitude of the averaged IPSC for each cell by its τ_W._ The total IPSC charge delivered was the mean IPSC charge multiplied by the arithmetic IPSC frequency. To measure tonic GABA_A_ currents the mean holding current at V_h_ –70 mV in the absence and presence of drugs was measured by fitting a single Gaussian function to all-points histograms constructed from five one second long sampling periods (Bright and Smart, 2013). The holding current for each condition was measured as the mean of the Gaussian distribution and the root mean square (RMS) noise was the standard deviation of the distribution. To account for the hierarchical structure of the data and non-independencies within it (see Results; Fig. 1*E*), the amplitudes and instantaneous frequencies of IPSCs in DGGCs and PV^+^-INs were analyzed using a linear mixed effects (LMEs) model constructed in the open-source statistical software environment R (R Core Team, 2018) using the lme4 module (Bates et al., 2015). The model used for the analysis was a random intercept model including a single fixed effect (genotype: WT vs Cyfip1^+/–^) and two random effects accounting for variation between cells and between mice. The linearity and homoskedasticity of the data were confirmed by plotting the residuals obtained from the fitted model. Data obtained for both IPSC amplitudes and instantaneous frequencies we found to follow lognormal distributions. LME models were therefore fit to the natural logarithm [ƒ(x) = ln(x), x > 0] transformed IPSC amplitude and instantaneous frequency data which followed approximately normal distributions (Figs. 1, 3). Subsequently, mean (μ) IPSC amplitude (in pA) and frequency (in Hz) for each genotype were obtained by inverting the intercept values obtained from the LME models (fitted to log-transformed data) using the exponential function [ƒ^−1^(μ) = e^μ^]. Thus, the mean values reported for IPSC amplitude and instantaneous frequency are the geometric rather than arithmetic means of the data. The geometric mean of the log-normally distributed data represents the “central tendency” of the data better than the arithmetic mean and gives a more accurate measure of the typical event without the distorting effect of the relatively small number of large amplitudes or high instantaneous frequencies IPSCs that skew the distribution. The geometric mean IPSC amplitudes and instantaneous frequencies obtained using this approach are reported with their 95% confidence intervals (95% CIs) which are asymmetric around the mean. Upper and lower confidence limits were calculated as the exponential [ƒ(CI) = e^CI^] of the mean plus or minus 1.96 times the standard error of the intercept vales obtained from the LME model fits. The effect of genotype on IPSC amplitude/frequency was tested using the “anova()” function in R (likelihood ratio test) to compare our model with a null model which excluded the fixed effect. Unless otherwise indicated, n refers to the number of cells recorded under each condition. For each condition cells were sampled from a minimum of 3 different non-littermate mice for each genotype. Data analysis was performed using pClamp 10 (Molecular Devices), Prism 5 (GraphPad) software and R (https://www.R-project.org/). Statistical testing was by paired/unpaired *t* test or one-way ANOVA where appropriate and as indicated in text.

For ISH, qRT-PCR and immunoblotting techniques, outliers >2.5 SD from the mean were removed and significance was determined by one-way or two-way ANOVA using SPSS software (IBM, v.20). *Post hoc* Dunnett’s multiple comparison procedure was applied to data which surpassed significance threshold (α = 0.05) in ANOVA, to determine specific group differences. Detailed statistical analyses can be found in the figure legends. All values are given as mean ± SEM, with the exception of qRT-PCR data given as mean ± SD.

## Results

### GABAergic inhibition is unaltered in DGGCs of *Cyfip1* ^+/–^ mice

We performed whole-cell voltage clamp recordings from DGGCs to determine the effects of *Cyfip1* haploinsufficiency on GABAergic inhibition. To confirm that all DGGCs we recorded from were fully mature neurons, and not from immature adult born granule cells, we first performed current clamp recordings on a subset (*n* = 4) of DGGCs. This revealed, as shown previously, that cells with mature morphology and dendrites projecting into the outer molecular layer (ML; Fig. 1*A*) also displayed firing properties and input resistances characteristic of mature cells (<300 MΩ; Fig. 1*B*). Thus, in subsequent voltage clamp experiments, we considered recorded cells to be fully mature granule cells based on inspection of their morphology after filling with Alexa Fluor 594. First, we measured properties of synaptic inhibition in DGGCs by recording sIPSCs. In control cells, from WT mice, our recordings revealed that sIPSCs properties were similar to those reported for DGGC in previous studies, consistent with a large proportion of their inhibitory input coming from local fast-spiking interneurons (Fig. 1*C*; Bartos et al., 2001; Nusser and Mody, 2002; Chandra et al., 2006). First, we analyzed the effect of genotype on IPSC amplitudes in DGGCs. We found that the amplitudes of sampled IPSCs in both WT and *Cyfip1^+/–^* DGGCs were approximately log-normally distributed (Fig. 1*E*). Thus, for subsequent analysis we used the corresponding log-transformed data which followed a normal distribution (Fig. 1*F*). Since many individual IPSCs were recorded from each neuron (i.e., repeated measures) and several neurons recorded from individual mice (Fig. 1*E*,*F*) it is clear our data violates the critical assumption of independence required for linear modeling. Therefore, we chose to fit the log-transformed IPSC amplitudes, pooled from all recorded DGGCs (WT: 9494 IPSCs from 25 cells/five mice, *Cyfip1^+/–^*: 4948 IPSCs from 14 cells/four mice), using a LMEs model. Using LME models allows us to analyze the effects of genotype across our entire sample of IPSCs while accounting for the non-independencies that result from the hierarchical structure of our data. An example of the hierarchical structure within the data can be seen in in the scatterplot shown in Figure 1*E*, which shows individual IPSCs grouped by neuron, by mouse and by genotype. In our analysis, the LME models we have used account for the random variation across individual neurons and individual mice allowing us to isolate the effect of genotype on the dependent variable (IPSC amplitude/frequency). Using this approach we found that genotype did not significantly affect the amplitude of IPSCs in DGGC (χ^2^(1) = 0.06, *p* = 0.80) with WT IPSCs having a mean amplitude of 25.7 pA (95% CI: 21.1, 31.2) and *Cyfip1^+/–^* IPSCs having an amplitude of 26.3 pA (95% CI: 22.6, 30.6). Moreover, we found no significant differences in the weighted decay time constant (τ_W_; *p* = 0.70, unpaired *t* test; Table 1; Fig. 1*C*,*D*) and charge transfer (*p* = 0.95, unpaired *t* test; Table 1) for averaged IPSCs between *Cyfip1^+/–^* mice and their WT counterparts. Similarly, we also found that the instantaneous frequencies (1/inter-IPSC interval) of both WT and *Cyfip1^+/–^* IPSCs were log-normally distributed (Fig. 1*G*) and that their corresponding log-transformed values were normally distributed (Fig. 1*H*). Fitting an LME model revealed that genotype had no significant effect on IPSC instantaneous frequency (χ^2^(1) = 0.67, *p* = 0.41) with WT IPSCs having a mean frequency of 7.42 Hz (95% CI: 6.44, 8.55) and *Cyfip1^+/–^* IPSCs having a frequency of 6.86 Hz (95% CI: 5.74, 8.18), respectively. Thus, we find that haploinsufficiency of *Cyfip1* does not impair phasic GABAergic inhibition in DGGC of the mouse hippocampus.

**Table 1:** IPSC properties in DGGCs and PV^+^-INs of WT and *Cyfip1^+/–^* **mice**

	*n*	Peak amplitude (pA)	Weighted decay (ms)	Frequency (Hz)	Charge transfer (fC)	Total IPSC charge (pC)
DGGC						
WT	25	29.5 ± 1.8	4.4 ± 0.1	6.3 ± 0.4	128.8 ± 8.3	0.87 ± 0.1
*Cyfip1^+/-^*	14	29.7 ± 2.2	4.3 ± 0.1	5.9 ± 0.5	127.9 ± 10.3	0.76 ± 0.1
PV^+^-INs						
WT	18	44.9 ± 4.2	1.8 ± 0.2	25.1 ± 2.1	82.2 ± 11.5	2.28 ± 0.5
*Cyfip1^+/-^*	13	44.0 ± 4.0	2.0 ± 0.2	27.9 ± 2.4	87.8 ± 9.9	2.54 ± 0.4

*n* = number of neurons from a minimum of three mice per genotype.

**Figure 2.**
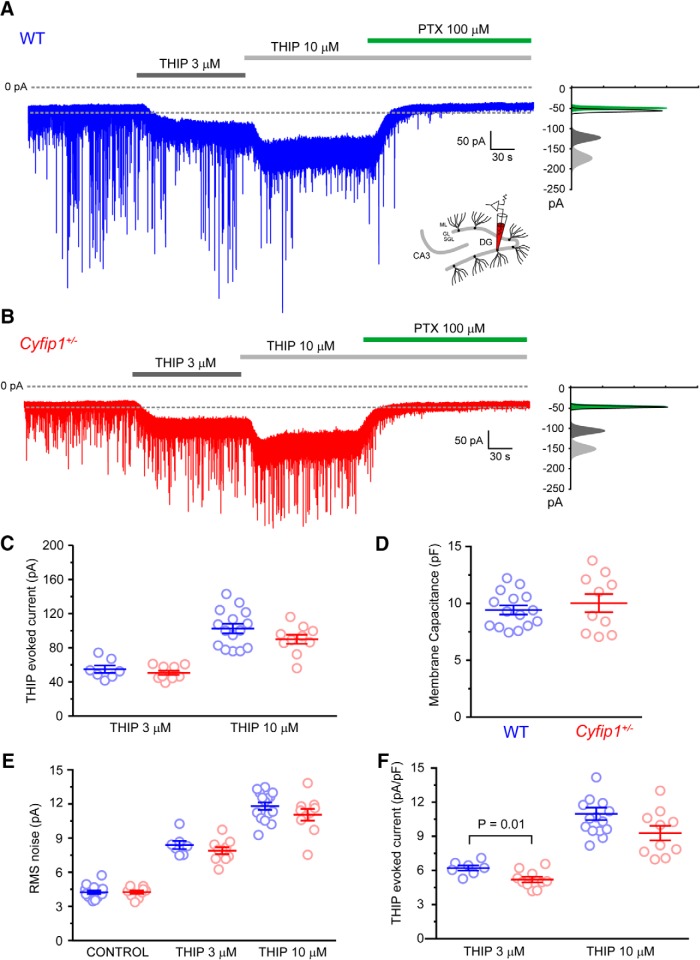
THIP-evoked currents in DGGC of WT and *Cyfip1^+/–^* mice. ***A***, Trace showing PTX-sensitive concentration-dependent THIP-evoked currents in a DGGC from a WT mouse. All-points histogram illustrates the shift in holding current and RMS noise induced by THIP and PTX. ***B***, As in ***A*** for DGGC in *Cyfip1^+/–^* mouse. ***C***, Scatter plot summarizing the THIP-evoked currents in WT (blue) and *Cyfip1^+/–^* DGGC. ***D***, Scatter plot summarizing membrane capacitance of WT (blue) and *Cyfip1^+/–^* DGGC. ***E***, Scatter plot summarizing the THIP-evoked changes in RMS noise in WT (blue) and *Cyfip1^+/–^* DGGC. ***F***, Scatter plot summarizing the normalized (pA/PF) THIP-evoked current in WT (blue) and *Cyfip1^+/–^* DGGC.

However, as discussed earlier, FMRP can reduce tonic inhibition through mechanisms that are not dependent on changes in presynaptic neurotransmitter release, in particular, a direct postsynaptic reduction of the expression of GABA_A_R δ-subunits (D’Hulst et al., 2006; Curia et al., 2009; Zhang et al., 2017). To test whether the loss of one *Cyfip1* allele and reduction in expression of the FMRP binding partner CYFIP1, which mimics microdeletion at locus 15q11.2 in humans, produces a similar reduction in GABA_A_R δ-subunit-dependent tonic inhibition as observed in FXS, we evoked currents using the δ-subunit-selective drug THIP (Gaboxadol). We measured THIP-evoked currents using both the drug-induced shift in holding current and difference in RMS noise as the latter has been suggested to be more sensitive to detecting small differences in tonic inhibition (Bright and Smart, 2013). To evoke sufficiently large currents to allow us to easily compare between genotypes we used THIP at concentrations of 3 and 10 μM. At 3 μM, THIP is largely selective for high affinity δ-subunit-containing receptors, whereas at 10 μM it may also substantially activate δ-subunit-lacking receptors (i.e., αβ pentamers; Meera et al., 2011). This approach allowed us to investigate differences in both of the potential pools of extrasynaptic receptors in these cells across genotypes. In DGGCs from WT animals, bath application of THIP produced a concentration dependent increase in the current required to hold the cell at –70 mV (ΔI_THIP_; WT: ΔI_THIP 3 μM_: 54.8 ± 4.4 pA, *n* = 7; ΔI_THIP 10 μM_: 102.5 ± 5.6 pA, *n* = 15; Fig. 2*A*,*C*) and a corresponding increase in RMS noise (WT: RMS_control_: 4.24 ± 0.15 pA, *n* = 15; RMS_THIP 3 μM_: 8.39 ± 0.35 pA, *n* = 7; RMS_THIP 10 μM_: 11.81 ± 0.33 pA, *n* = 15; Fig. 2*A*,*E*) indicating increased opening of δ-subunit-containing eGABA_A_Rs. The THIP-evoked currents were completely blocked by the GABA_A_ channel blocker PTX (100 μM) confirming their GABAergic nature (Fig. 2*A*). We observed a very slight, but not significant, reduction in both the magnitude of ΔI_THIP_ (*Cyfip1^+/–^*: ΔI_THIP 3 μM_: 50.6 ± 2.5 pA, *n* = 10, *p* = 0.39, ΔI_THIP 10 μM_: 90.0 ± 5.3 pA, *n* = 10, *p* = 0.14, unpaired *t* test; Fig. 2*B*,*C*) and RMS noise (*Cyfip1^+/–^*: RMS_control_: 4.25 ± 0.14 pA, *n* = 10, *p* = 0.96; RMS_THIP 3 μM_: 7.90 ± 0.31 pA, *n* = 10, *p* = 0.31; RMS_THIP 10 μM_: 11.06 ± 0.52 pA, *n* = 10, *p* = 0.21; Fig. 2*B*,*E*) in *Cyfip1^+/–^* compared to WT DGGCs. To confirm that the lack of observed difference in ΔI_THIP_ was not due to a compensatory change resulting from a genotype dependent change in dendritic size or complexity we normalized the evoked currents per pF of membrane capacitance (C_m_). Although we observed no significant (*p* = 0.47, unpaired *t* test) difference in C_m_ between WT (9.4 ± 0.4 pF, *n* = 15) and *Cyfip1^+/–^* (10.0 ± 0.8 pF, *n* = 10) DGGCs (Fig. 2*D*), normalization of ΔI_THIP_ to C_m_ revealed a trend toward a reduction in THIP-evoked currents in *Cyfip1^+/–^* DGGC (ΔI_THIP 3 μM_: 5.2 ± 0.2 pA/pF, *n* = 10; ΔI_THIP 10 μM_: 9.3 ± 0.6 pA/pF, *n* = 10) compared to WT DGGC (ΔI_THIP 3 μM_: 6.2 ± 0.2 pA/pF, *n* = 7, *p* = 0.01; ΔI_THIP 10 μM_: 11.0 ± 0.5 pA/pF, *n* = 15, *p* = 0.06; Fig. 2*F*), although the difference was small and only statistically significant for the lower concentration of THIP. Finally, to determine whether changes in tonic inhibition might occur later in life, we performed recordings from a small number of adult WT and *Cyfip1^+/–^* mice (*n* = 2 mice per genotype). Similarly to the findings in adolescent mice, we found currents evoked by 3 μM THIP were not significantly different between genotypes in DGGCs of older mice (WT: ΔI_THIP 3 μM_: 62.9 ± 5.9 pA, *n* = 9; *Cyfip1^+/–^*: ΔI_THIP 3 μM_: 70.3 ± 7.1 pA, *n* = 9, *p* = 0.44, unpaired *t* test). Thus, overall, we conclude that haploinsufficiency of *Cyfip1* does not significantly alter GABAergic inhibition in DGGCs of the mouse hippocampus.

### GABAergic inhibition is unaltered in GCL PV^+^-INs of *Cyfip1^+/–^* mice

**Figure 3.**
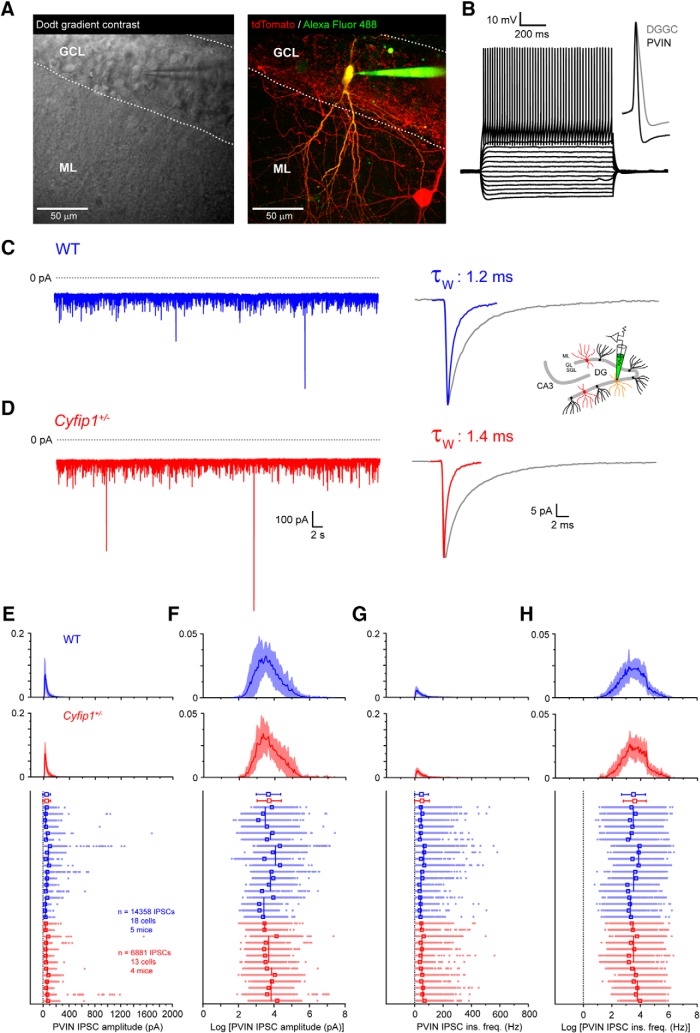
IPSCs in GCL PV^+^-INs of WT and *Cyfip1^+/–^* mice. ***A***, Dodt gradient contrast image of a horizontal section of the hippocampus. Dashed lines illustrate the border between the ML, GCL, and SGZ. An example of PV^+^-INs in the DG GCL identified by expression of the red fluorescent protein tdTomato filled via the recording electrode with Alexa Fluor 488 (right). ***B***, Traces depicting typical electrophysiological properties of a GCL PV^+^-INs. Inset shows the shorter half-width of a typical PV^+^-IN action potential compared to that of a typical DGGC. ***C***, Voltage clamp recording showing IPSCs from PV^+^-INs of WT mouse and the averaged IPSC from this cell. Inset schematic shown here and elsewhere signifies data recorded PV^+^-INs. ***D***, A typical voltage clamp recording showing IPSCs from PV^+^-INs of *Cyfip1^+/–^* mouse and the mean IPSC from this cell. ***E***, Histograms showing the mean (red/blue lines) and standard deviation (light blue/red shading) of the log –normal distribution of all IPSC amplitudes recorded in WT and *Cyfip1^+/–^* PV^+^-INs. Scatter plot shows the amplitude of individual IPSCs group by cell, mouse, and genotype. Individual IPSCs from each cell are shown as aligned dots (light blue/red) with the mean IPSC amplitude for each cell shown as an unfilled superimposed square (red/blue). Varying length vertical lines (blue/red) represent the mean IPSC amplitude for each animal with the length of the bar illustrating the number of recorded neurons from each mouse. Red/blue symbols (top) are the arithmetic mean and standard deviation of the complete dataset for each genotype. ***F***, As in ***E*** but for log-transformed IPSC amplitude data. ***G***, As in ***E*** but for IPSC instantaneous frequency data. ***H***, As in ***E*** but for log-transformed IPSC frequency data.

As well as DGGCs, detailed immunohistochemical studies have identified that interneurons of the DG also express GABA_A_R δ-subunits. In particular, PV^+^-INs found in the GCL and SGZ of the DG have 100% co-expression of parvalbumin and GABA_A_R δ-subunits (Milenkovic et al., 2013). However, it remains to be demonstrated that PV^+^-INs in these layers express functional δ-subunit-dependent tonic GABAergic currents. Since PV^+^-INs have been previously implicated as key targets in neuropsychiatric disorders, we tested for the functional presence of eGABAergic inhibition in these cells in *Cyfip1^+/–^* and WT mice. To do this, we crossed a PV^+^-Cre mouse with a tdTomato reporter mouse line and subsequently the *Cyfip1^+/–^* mouse (PV^+^TdCyfip1) and made targeted patch clamp recordings from GCL PV^+^-INs (Fig. 3*A*). Current-clamp recordings from GCL PV-INs (*n* = 3) showed that these PV^+^ cells had firing properties, typical of fast-spiking basket cells including high frequency firing and action potentials with characteristic short half-widths (Fig. 3*B*). First, we compared phasic GABAergic inhibition in GCL PV^+^-INs in *Cyfip1^+/–^* and WT mice. Consistent with previous studies, IPSCs in PV^+^-INs decayed more rapidly than those recorded in DGGC with significantly shorter decay time constants (τ_W_; WT: DGGC: 4.4 ± 0.1 ms, *n* = 25, WT: PV^+^-IN: PV^+^-IN 1.8 ± 0.2 ms, *n* = 18, *p* < 0.0001, unpaired *t* test; Table 1; Fig. 3*C*,*D*). This can be seen clearly in the overlaid traces shown in Figure 3*C*,*D*and is consistent with a higher level of expression of α_1_-subunits in PV^+^-INs compared to DGGCs (Bartos et al., 2001, 2002). Similarly, to DGGCs, IPSC amplitudes (Fig. 3*E*) and instantaneous frequencies (Fig. 3*G*) in PV^+^-INs were found to be log-normally distributed. Therefore, we fit LME models to the log-transformed data values, which were approximately normally distributed (Fig. 3*F*,*H*), to account for the hierarchical structure of the data as depicted in the scatterplots in Figure 3*F*,*H*. As for DGCGs, we found that genotype had no effect on IPSC amplitudes in PV^+^-INs (χ^2^(1) = 0.03, *p* = 0.86) with WT IPSCs (*n* = 14,358 IPSCs from 18 cells/five mice) having a mean amplitude of 39.3 pA (95% CI: 30.3, 50.9) and *Cyfip1^+/–^* IPSCs (*n* = 6881 IPSCs from 13 cells/four mice) having a mean amplitude of 40.2 pA (95% CI: 33.0, 49.0). We also found no significant difference in the weighted decay time constant (τ_W_; *p* = 0.32, unpaired *t* test; Table 1; Fig. 3*C*,*D*) and charge transfer (*p* = 0.73, unpaired *t* test; Table 1) for IPSCs between *Cyfip1^+/–^* mice and WT littermates. In comparison to DGGC, PV^+^-INs had an ∼4-fold higher arithmetic mean IPSC frequency (Table 1; Fig. 3*C*,*F*). Fitting an LME model to the log-transformed PV^+^-IN IPSC instantaneous frequency data revealed a mean WT IPSC instantaneous frequency of 32.1 Hz (95% CI: 25.1, 41.0) compared to a mean *Cyfip1^+/–^* IPSC frequency of 35.9 Hz (95% CI: 29.9, 43.3). Genotype did not significantly (χ^2^(1) = 0.81, *p* = 0.37) affect IPSC instantaneous frequency in PV^+^-INs. Thus, as with DGGCs, we conclude that *Cyfip1* haploinsufficency does not change the properties of IPSCs in PV^+^-INs of the DG.

**Figure 4.**
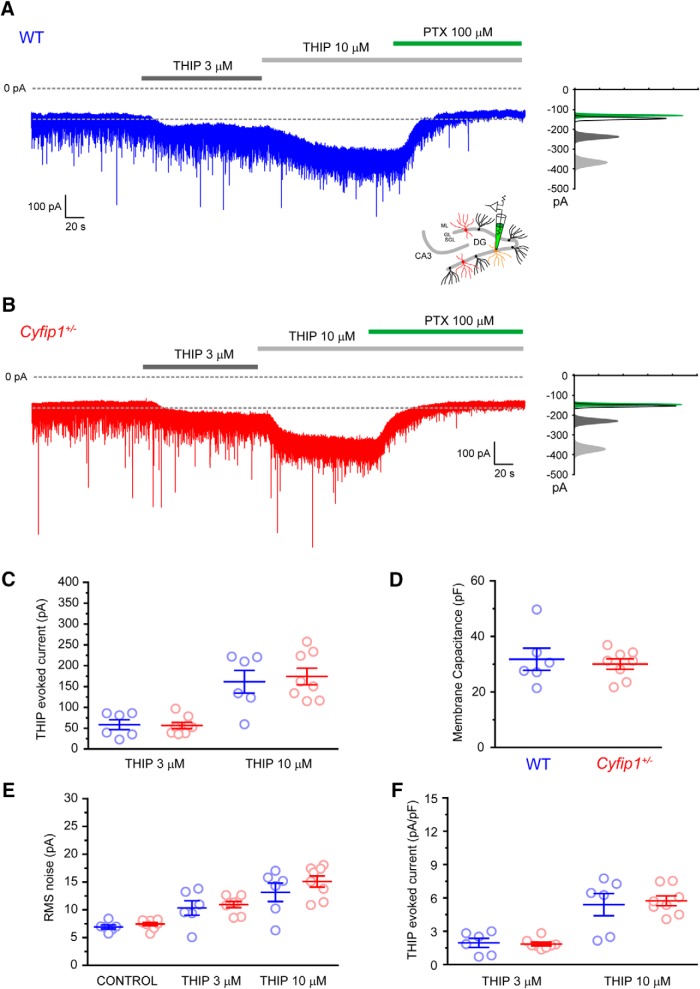
THIP-evoked currents in PV^+^-INs of WT and *Cyfip1^+/–^* mice. ***A***, Trace showing PTX-sensitive concentration-dependent THIP-evoked currents in a PV^+^-IN from a WT mouse. All-points histogram illustrates the shift in holding current and RMS noise induced by THIP and PTX. ***B***, As in ***A*** for PV^+^-INs in *Cyfip1^+/–^* mouse. ***C***, Scatter plot summarizing the THIP-evoked currents in WT (blue) and *Cyfip1^+/–^* PV^+^-INs. ***D***, Scatter plot summarizing membrane capacitance of WT (blue) and *Cyfip1^+/–^* PV^+^-INs. ***E***, Scatter plot summarizing the THIP-evoked changes in RMS noise in WT (blue) and *Cyfip1^+/–^* DGGC. ***F***, Scatter plot summarizing the normalized (pA/PF) THIP-evoked current in WT (blue) and *Cyfip1^+/–^* DGGC.

Unlike DGGCs, it remains to be demonstrated whether PV-INs in the GCL of the DG express functional δ-subunit-containing eGABA_A_Rs and have tonic GABAergic inhibition. Therefore, we next used THIP to demonstrate the presence of these receptors in molecularly identified GCL PV^+^-INs and to compare their relative activation in *Cyfip1^+/–^* and WT cells. In WT PV^+^-INs, THIP produced a concentration-dependent PTX-sensitive increase in holding current (WT: ΔI_THIP 3 μM_: 58.5 ± 11.9 pA, *n* = 6; ΔI_THIP 10 μM_: 161.6 ± 27.2 pA, *n* = 6; Fig. 4*A*,*C*) that was accompanied by a marked increase in RMS noise (WT: RMS_control_: 6.92 ± 0.36 pA, *n* = 6; RMS_THIP 3 μM_: 10.33 ± 1.29 pA, *n* = 6; RMS_THIP 10 μM_: 13.15 ± 1.66 pA, *n* = 6; Fig. 4*A*,*E*) demonstrating the presence of δ-subunit-containing eGABA_A_Rs. However, we found no significant difference in the magnitude of THIP-evoked currents (*Cyfip1^+/–^*: ΔI_THIP 3 μM_: 56.6 ± 7.6 pA, *n* = 8, *p* = 0.71; ΔI_THIP 10 μM_: 174.2 ± 19.9 pA, *n* = 8, *p* = 0.89, unpaired *t* test) or changes in RMS noise (*Cyfip1^+/–^*: RMS_control_: 7.44 ± 0.30 pA, *n* = 8, *p* = 0.29; RMS_THIP 3 μM_: 10.94 ± 0.55 pA, *n* = 8, *p* = 0.64; RMS_THIP 10 μM_: 15.09 ± 1.00 pA, *n* = 8, *p* = 0.31, unpaired *t* test; Fig. 4*B*,*E*) in *Cyfip1^+/–^* PV^+^-INs compared to WT controls. We found, as measured by cell capacitance, that GCL PV^+^-INs were approximately three times larger than DGGCs but that there was no difference in cell size between genotypes (WT: 31.8 ± 4.0 pF, *n* = 6, *Cyfip1^+/–^*: 30.1 ± 1.87 pF, *n* = 8, *p* = 0.68, unpaired *t* test; Fig. 4*D*). Consequently, when normalized to cell capacitance no difference in THIP-evoked currents in WT (ΔI_THIP 3 μM_: 2.0 ± 0.4 pA/pF, *n* = 6; ΔI_THIP 10 μM_: 5.4 ± 1.0 pA/pF, *n* = 6) compared to *Cyfip1^+/–^* (ΔI_THIP 3 μM_: 1.8 ± 0.2 pA/pF, *n* = 8, *p* = 0.80, ΔI_THIP 10 μM_: 5.7 ± 0.4 pA/pF, *n* = 8, *p* = 0.73, unpaired *t* test) PV^+^-INs was observed. Thus, similarly to DGGCs, haploinsufficiency of *Cyfip1* does not significantly alter GABAergic inhibition in GCL PV^+^-INs of the mouse hippocampus.

### mRNA expression and protein levels of δ and other key GABA_A_R subunits in hippocampus

**Figure 5.**
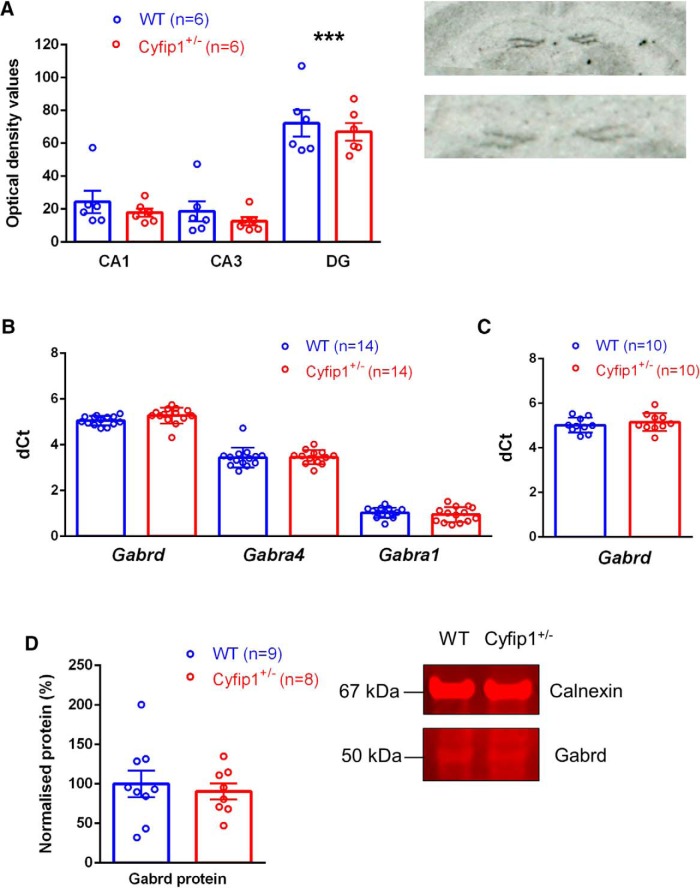
Molecular assessment of δ and other key GABA_A_ subunits in WT and *Cyfip1^+/–^* mice. ***A***, mRNA expression of δ GABA_A_ subunit (*Gabrd*) in hippocampal subfields (CA1, CA3, DG) of WT and *Cyfip1^+/–^* adult mice were measured by ISH and given as absolute mean optical density values ± SEM (*n* = 6 per genotype). Once ANOVA revealed a main effect for brain region, Dunnett’s test was used for *post hoc* analysis to determine the sources of significance (DG vs CA1 and DG vs CA3, ***p < 0.0001). Representative autoradiographs show two coronal WT mouse brain sections hybridized with an oligonucleotide probe targeting *Gabrd*. ***B***, Expression of GABA_A_ δ-, α_4_-, and α_1_-subunits (equivalent to *Gabrd*, *Gabra4*, and *Gabra1* genes) were measured by qRT-PCR mRNA expression in adult whole hippocampus and given as mean delta Ct values ± SD relative to two housekeeping genes, *Gapdh* and *Hprt* (*n* = 14 per genotype, 1 outlier removed in *Gabra4* data). ***C***, As in ***B***, qRT-PCR was used to measure *Gabrd* mRNA expression in juvenile WT and *Cyfip1^+/–^* mice (*n* = 10 per genotype). ***D***, Hippocampal protein levels of δ GABA_A_ were measured by immunofluorescent Western blotting in WT and *Cyfip1^+/–^* mice. *Cyfip1^+/–^* densitometric data given relative to the average of all WT samples (100%) and normalized to protein loading control, calnexin (WT: *n* = 9, *Cyfip1^+/–^*: *n* = 8). Representative immunofluorescent blot shows Gabrd and calnexin bands in WT and *Cyfip1^+/–^* mouse samples.

To confirm our electrophysiological findings, we first measured the expression level of the GABA_A_R δ-subunit (encoded by *Gabrd*) in adult WT and *Cyfip1^+/–^* mice. Using ISH techniques, we measured GABA_A_R δ-subunit mRNA expression in three major subfields of the hippocampus including the DG (CA1, CA3, DG; Fig. 5*A*). We confirmed, as shown previously (Pirker et al., 2000; Zheng et al., 2009; Milenkovic et al., 2013; Fritschy and Panzanelli, 2014), that the majority of GABA_A_R δ-subunit expression occurs in the DG with little δ-subunit expression in the CA1 and CA3 hippocampal subfields (brain region: *F*_(2,30)_ = 55.370, *p* = 0.0001, two-way ANOVA; *post hoc* Dunnett’s test show DG v CA1 and DG v CA3 were both *p* = 0.0001). Significantly, we observed no influence of *Cyfip1* haploinsufficiency on GABA_A_R δ-subunit mRNA expression and its distribution across the hippocampus (genotype: *F*_(1,30)_ = 1.656, *p* = 0.208; genotype × brain region *F*_(2,30)_ = 0.006, *p* = 0.994, two-way ANOVA). The lack of change in GABA_A_R δ-subunit expression was confirmed by complementary qRT-PCR techniques performed in the whole hippocampus of adult WT and *Cyfip1^+/–^* mice (*Gabrd* exp: *F*_(1,26)_ = 2.526, *p* = 0.124, one-way ANOVA; Fig. 5*B*).

We next measured the mRNA expression of GABA_A_R α_4_-subunits (*Gabra4*), which are co-expressed with δ-subunits in the majority, if not all, eGABA_A_R in DGGC (Sun et al., 2004; Chandra et al., 2006). Consistent with a lack of change in δ-subunit expression, we found no significant difference in the α_4_-subunit expression in hippocampus of adult WT and *Cyfip1^+/–^* mice (*Gabra4* exp: *F*_(1,25)_ = 0.105, *p* = 0.749, one-way ANOVA, 1 outlier removed; Fig. 5*B*). This was in agreement with unaltered GABAergic inhibition in DGGC (Figs. 1, 2). Furthermore, we assessed the expression of GABA_A_R α_1_-subunits (*Gabra1*) in WT and *Cyfip1^+/–^* mice, since these subunits can also form high affinity GABA_A_R with δ-subunits (Glykys et al., 2007; Karim et al., 2013) and are highly expressed in GCL PV^+^-INs (Milenkovic et al., 2013). In line with our electrophysiological observations (Fig. 3), which showed no difference in tonic inhibition in PV^+^-INs, we found no difference in α_1_-subunit expression in adult WT and *Cyfip1^+/–^* mice (*Gabra1* exp: *F*_(1,26)_ = 0.849, *p* = 0.365, one-way ANOVA; Fig. 5*B*).

Lastly, to complement the majority of our electrophysiological work, which was conducted in adolescent rather than adult mice, we extended our analysis of GABA_A_R δ-subunit expression into adolescent WT and *Cyfip1^+/–^* mice. Our data confirmed that hippocampal GABA_A_R δ-subunit mRNA expression (*Gabrd* mRNA exp: *F*_(1,18)_ = 0.545, *p* = 0.470, one-way ANOVA; Fig. 5*C*) and protein levels were also unaffected by *Cyfip1* haploinsufficiency (Gabrd protein: *F*_(1,15)_ = 0.221, *p* = 0.221, one-way ANOVA; Fig. 5*D*) in adolescent mice.

### Tonic inhibition in GCL PV^+^-INs involves α_1_-subunit-containing eGABA_A_Rs

**Figure 6.**
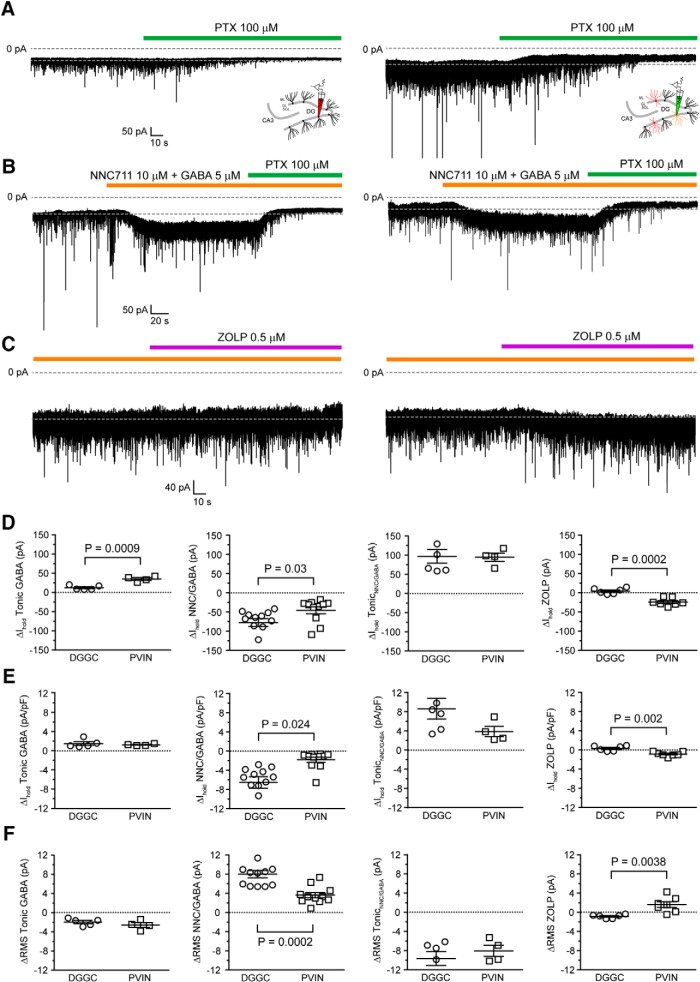
Tonic inhibition in PV^+^-INs is sensitive to extracellular GABA concentration and modulated by the α_1_-selective ligand zolpidem. ***A***, Example traces showing the ∼3-fold larger basal tonic GABA current in PV^+^-INs compared to DGGCs revealed by application of the GABA_A_ antagonist PTX. ***B***, Example traces showing the modulation of tonic inhibition in PV^+^-INs and DGGCs by the elevation of extracellular GABA concentration using NNC711 (10 μM) and GABA (5 μM). ***C***, Example traces showing the modulation of tonic inhibition in PV^+^-INs, but not DGGCs, by the α_1_-selective ligand zolpidem. ***D***, Scatter plots summarizing the changes in holding current (ΔI_hold_) in DGGCs and PV^+^-INs reflecting the basal tonic GABAergic inhibition, the current induced by NNC/GABA, the tonic GABA current in NNC/GABA and the current induced by zolpidem. ***E***, As in ***D*** for normalized currents (pA/pF). ***F***, As in ***D*** for changes in RMS noise.

Although we found no significant difference in THIP-evoked currents in molecularly defined GCL PV^+^-INs between *Cyfip1^+/–^* and WT mice, our data demonstrates for the first time to our knowledge, the presence of functional tonic GABAergic inhibition in these GCL interneurons. Consequently, we decided to further characterize the tonic GABA currents in these interneurons and compare them with their neighboring granule cells. Therefore, we first measured basal tonic current in both PV^+^-INs and DGGCs in WT mice under our *in vitro* conditions using the GABA channel blocker PTX (100 μM). In DGGCs, application of PTX produced a significant reduction in the holding current (Table 2) of 12 ± 2.5 pA (Fig. 6*A*,*D*) which was accompanied by a reduction in RMS noise of 1.98 ± 0.33 pA (Fig. 6*A*,*F*). The tonic current we recorded under basal conditions in DGGC was similar to that previously reported for these cells (Nusser and Mody, 2002; Chandra et al., 2006; Wlodarczyk et al., 2013). On the other hand, basal tonic currents in PV^+^-INs (34.9 ± 3.5 pA, *n* = 4) were ∼3-fold greater than those we observed in DGGCs (Table 2; Fig. 6*A*,*D*). However, when normalized to cell capacitance, the tonic current density was not significantly different in PV^+^-INs (1.21 ± 0.09 pA/pF, *n* = 4) compared to DGGCs (1.45 ± 0.38 pA/pF, *n* = 5, *p* = 0.61, unpaired *t* test; Fig. 6*E*). Interestingly, when the fraction of total GABAergic inhibition provided by tonic inhibition versus phasic IPSCs is compared between DGGCs and PV^+^-INs, we find remarkable similarity between the cell types. The mean total charge was calculated as the sum of the total charge provided by IPSCs (Table 1) plus the charge provided by tonic inhibition. For DGGCs, IPSCs provided 6.7% of the total inhibitory charge (0.87 pC of 12.97 pC total charge), and for PV-INs, IPSCs provided 6.1% of the total inhibitory charge (2.28 pC of 37.18 pC total charge). The similarity between these values and their similarity to the fraction of inhibitory charge provided by tonic inhibition in other cell types (Brickley et al., 1996; Rossi et al., 2003; Cope et al., 2005) suggests that expression of eGABA_A_R might be regulated to constrain the ratio of tonic to phasic inhibition within fairly narrow bounds across both inhibitory and excitatory neurons and this might be critical for stability of neuronal input-output transformations (Pavlov et al., 2009).

**Table 2.** Pharmacologically induced changes in holding current in DGGCs and PV^+^-INs

Basal tonic GABA current					
	*n*	I_hold_ control (pA)	I_hold_ PTX (pA)	RMS control (pA)	RMS PTX (pA)
DGGC	5	–68.4 ± 9.7	–56.3 ± 8.3**	5.2 ± 0.4	3.2 ± 0.2**
PVIN	4	–85.9 ± 8.6	–51.1 ± 9.8**	7.6 ± 0.8	5.0 ± 0.8*
NNC- and GABA-evoked current					
	*n*	I_hold_ control (pA)	I_hold_ NNC/GABA (pA)	RMS control (pA)	RMS NNC/GABA (pA)
DGGC	12	–67.2 ± 3.9	–140.3 ± 11.5****	4.6 ± 0.1	12.6 ± 0.8****
PVIN	11	–147.8 ± 20.8	193.4 ± 21.1***	8.1 ± 0.7	11.7 ± 0.7****
Tonic GABA current in NNC and GABA					
	*n*	I_hold_ NNC/GABA (pA)	I_hold_ PTX (pA)	RMS NNC/GABA (pA)	RMS PTX (pA)
DGGC	6	–155.7 ± 20.7	–59.2 ± 6.9**	13.2 ± 1.5	3.6 ± 0.8**
PVIN	4	–148.6 ± 23.6	–54.5 ± 14.8**	14.2 ± 1.0	6.1 ± 0.2**
Zolpidem-evoked current					
	*n*	I_hold_ NNC/GABA (pA)	I_hold_ ZOLP (pA)	RMS NNC/GABA (pA)	RMS ZOLP (pA)
DGGC	6	–118.0 ± 8.9	–113.7 ± 7.1	11.8 ± 0.7	11.0 ± 0.7**
PVIN	7	–205.6 ± 30.2	–229.4 ± 28.6***	10.2 ± 0.4	11.8 ± 0.7*

*n* = number of neurons from a minimum of three mice per genotype. *p < 0.05, **p < 0.01, ***p < 0.001, ****p < 0.0001.

So far, we have shown that GCL PV^+^-INs are regulated by tonic GABAergic inhibition, as evidenced by their sensitivity to PTX, and that the eGABA_A_R underlying this tonic inhibition contain GABA_A_R δ-subunits as revealed by their sensitivity to the agonist THIP. However, previous histologic experiments have shown that PV^+^-INs have high expression of α_1_ GABA_A_R subunits and weaker expression of α_4_-subunits which predominate in DGGC (Sun et al., 2004; Chandra et al., 2006; Milenkovic et al., 2013). It has previously been shown that α_1_-subunits can form receptors with δ-subunits but that these receptors have a markedly lower affinity for GABA than α_4_ containing receptors (Karim et al., 2013). Therefore, we next compared how tonic inhibition in PV^+^-INs and DGGCs is changed by alterations in the extracellular GABA concentration. To do this we applied the GAT-1 inhibitor NNC-711 (10 μM) along with 5 μM GABA to elevate the concentration of GABA in the extracellular environment. In both cell types, bath application of NNC-711/GABA significantly increased the holding current (Table 2; Fig. 6*B*,*D*) and RMS noise (Table 2; Fig. 6*B*,*F*) compared to control demonstrating that tonic inhibition in PV^+^-INs, like DGGCs, is regulated by the GABA concentration in the extracellular space. Interestingly, the increase in tonic current was significantly larger in DGGCs compared to PV-INs (Fig. 6*B*,*D–F*), suggesting that the former express eGABA_A_R with higher affinity for GABA and are more sensitive to changes in extracellular GABA as would be expected for α_4_ containing receptors versus α_1_ containing ones (see Discussion).

Consequently, we next directly tested whether GCL PV^+^-INs express eGABA_A_R containing α_1_-subunits using the α_1_-selective non-benzodiazepine drug zolpidem. It is already established in DGGC that, although it modulates phasic inhibition by prolonging IPSC time constants, zolpidem does not modulate tonic inhibition due to its lack of effect at α_4_ containing eGABA_A_Rs (Nusser and Mody, 2002). We confirmed that application of 0.5 μM zolpidem did not significantly change the holding current (ΔI_hold_ ZOLP: –4.4 ± 3.1 pA) or RMS noise (ΔRMS ZOLP: –0.86 ± 0.17 pA) in DGGC (Table 2; Fig. 6*C–F*). On the other hand, we found that bath application of zolpidem to PV^+^-INs produced a significant increase in the holding current (ΔI_hold_ ZOLP: 23.9 ± 3.8 pA) and RMS noise (ΔRMS ZOLP: 1.61 ± 0.61 pA; Table 2; Fig. 6*C–F*). Thus, we conclude that molecularly defined PV^+^-INs in the DG GCL receive tonic GABAergic inhibition which is mediated by eGABA_A_Rs containing δ- and α_1_-subunits.

## Discussion

### Haploinsufficiency of *Cyfip1* does not alter GABAergic inhibition in DG

Loss of FMRP causes FXS and is associated with disrupted GABA signaling throughout the brain involving both pre- and postsynaptic mechanisms (Deng et al., 2011; Kang et al., 2017; Sabanov et al., 2017). A key binding partner of FMRP, CYFIP1, is encoded by the *CYFIP1* gene which is found at the 15q11.2 locus in humans. CNVs at this locus have been shown to significantly increase risk of development of neuropsychiatric and neurodevelopmental disorders including schizophrenia, autism spectrum disorder and intellectual disability. In this study, we set out to test the hypothesis that loss of a single copy of the gene encoding the FMRP-interacting protein *Cyfip1*, in a mouse model that mimics human disorders, would result in disruption of GABAergic inhibition in the hippocampus. We focused in particular on tonic GABAergic inhibition because disruption of this form of inhibition, resulting from reduced expression of the eGABA_A_R specific δ-subunit, has been previously demonstrated following loss of FMRP in a model of FXS, the *Fmr1* KO mouse (D’Hulst et al., 2006; Curia et al., 2009; Zhang et al., 2017). Contrary to our original hypothesis, however, and in contrast to findings from the FXS mouse, the results of our electrophysiological and histologic experiments demonstrate that, in DGGC and GCL PV^+^-INs, loss of a single copy of the *Cyfip1* gene is not sufficient to produce changes in either phasic or tonic GABAergic inhibition. However, we cannot rule out impaired GABAergic signaling in other subfields of the hippocampus (see later), or other brain regions, in this genetic model.

In this study, we investigated GABAergic inhibition in the DG of adolescent and adult WT and *Cyfip1^+/–^* mice. While the age of the adolescent mice (five to seven weeks) used in the majority of our experiments broadly matches the age of onset of schizophrenia symptoms in humans (Jones, 2013) it is noteworthy that the underlying pathologic causes of schizophrenia are neurodevelopmental and precede the presentation of symptoms, and diagnosis, by many years. Moreover, *Cyfip1* is a risk factor for both ASD and ID which are typically diagnosed in humans at a much younger juvenile age (Jones et al., 2014). While we did not investigate GABAergic inhibition earlier in postnatal development, our results show a lack of functional change in GABAergic inhibition in *Cyfip1* haploinsufficient adolescent mice that is in marked contrast to the effects seen in juvenile (Olmos-Serrano et al., 2011; Sabanov et al., 2017), adolescent/young adult (Curia et al., 2009) and adult (Centonze et al., 2008) mice with the complete loss of *Fmr1*. Our original hypothesis was that the convergent function of *Cyfip1* and *Fmr1,* through direct Cyfip1-FMRP interaction (Napoli et al., 2008), would regulate the translation of GABA_A_R signaling components (D’Hulst et al., 2006) and therefore influence inhibitory GABAergic function (Sabanov et al., 2017). Clearly, based on our new findings, this is not the case in the DG. Further, the disparate phenotypes observed in GABAergic inhibition in *Cyfip1* and *Fmr1* KO animal models of genetic psychiatric risk may simply relate to differing gene dosage (single copy loss of *Cyfip1* vs complete loss of *Fmr1*) and the subsequent levels of severity this has on the stoichiometry and/or function of the critically involved Cyfip1-Fmr1 complex. Thus, our findings suggest that either the interaction of CYFIP1 with FMRP is not critical in the ability of the latter to regulate eGABA_A_R expression or that a single copy of *Cyfip1* is sufficient to allow high enough protein expression to permit normal FMRP function. In the context of human pathology, this is of critical significance since CNVs at the 15q11.2 locus never result in *CYFIP1* homozygosity and in mice, *Cyfip1* homozygosity is embryonically lethal. Thus, we conclude that disrupted tonic GABAergic inhibition in the DG is unlikely to be a major contributor to the pathology of 15q11.2 CNV related disorders.

It should be noted that FMRP target mRNAs are thought to consist of 842 mRNAs that bind to FMRP, as part of a FMRP messenger ribonucleoprotein (mRNP) complex, in the mouse forebrain (Darnell et al., 2011). However, three separate studies have each found diverse FMRP target datasets that only partially overlap with Darnell’s FMRP mRNA targets (Brown et al., 2001; Miyashiro et al., 2003; Ascano et al., 2012). FMRP, in concert with CYFIP and as part of the functional FMRP-CYFIP1-eIF4E complex, represses protein translation of some of the FMRP target mRNAs (Napoli et al., 2008; Panja et al., 2014; Santini et al., 2017). Furthermore, Cyfip1 and FMRP proteins are known to form other neurobiological complexes and therefore partake in a range of distinctive functions. For instance, Cyfip1 forms part of the WAVE complex to regulate cytoskeletal dynamics (Schenck et al., 2001), and FMRP is a key hub protein, involved in chromatin and ion channel binding, for instance (Davis and Broadie, 2017), and so it might not be necessarily expected that phenotypes (including GABAergic signaling) of both models might closely map onto each other.

A critical feature of the DG is that it is one of only a few brain regions where new neurons are generated during adulthood. This process of neurogenesis, during which new DGGC are produced, takes place in the SGZ and GCL and is heavily modulated by GABAergic inhibition (Aimone et al., 2014). In particular, PV^+^-INs play an important role in the control of cellular proliferation and migration by release of GABA onto both neural progenitor cells (NPCs) and their progeny, new born DGGCs (Aimone et al., 2014; Song et al., 2012). It is therefore possible that more subtle or specific effects of the loss of *Cyfip1* may have a disruptive effect on GABAergic modulation of NPCs or immature DGGCs and thus impact on the neurogenic process although this remains to be investigated. Moreover, tonic inhibition mediated by δ-subunit-containing eGABA_A_R is not restricted to the DG of the hippocampus but plays a critical role in controlling cellular excitability in other hippocampal subfields and brain regions including the thalamus, cortex, cerebellum and striatum. In fact, Davenport and colleagues (2019) have recently examined the effect of complete Cyfip1 knock-out and GABA signaling in the CA1. Forebrain-specific Cyfip1 knock-out mice were shown to have increased mIPSCs in CA1 pyramidal cells, with an overall change in excitatory and inhibitory (E/I) balance. Intriguingly, the opposite effect was shown when Cyfip1 was overexpressed in cultured hippocampal neurons with decreased mIPSC amplitude, increased mEPSC frequency and an overall increase in E/I balance. Differences between our findings and those of Kittler might genuinely reflect pleiotropic effects of reduced Cyfip1 dosage across different hippocampal subfields (DG vs CA1). It should also be noted that Kittler’s findings derive from both the complete loss and *in vitro* overexpression of *Cyfip1*, while our data derives from the constitutive *Cyfip1* haploinsufficient mouse, modeling a CNV deletion that confers increased risk to a range of psychiatric disorders.

### Tonic inhibition in GCL PV^+^-INs

We found a lack of any genotype dependent functional difference in GABAergic signaling between WT and *Cyfip1^+/–^* mice in GCL PV^+^-INs. Our results do not rule out the possibility of genotype dependent differences in the expression of GABA_A_R subunits specifically in PV^+^-INs but do strongly suggest that any potential differences do not translate to deficits in functional inhibition in Cyfip1 haploinsufficient mice. Future studies, perhaps using single-cell RT-PCR or RNAseq techniques may provide further insight into the exact GABA_A_R subunit make-up in these cells following disruption of Cyfip1. However, despite the lack of functional effects of Cyfip1 on tonic GABAergic inhibition in GCL PV^+^-INs, our experiments have demonstrated, for the first time to our knowledge, that (1) PV^+^-INs in the GCL express functional eGABA_A_R, (2) that these receptors incorporate δ-subunits, and that (3) at least a proportion of the tonic inhibition in these cells is likely to be mediated through eGABA_A_R containing α_1_-subunits. Several lines of evidence lead us to these conclusions. In GCL PV^+^-INs, we found that the increase in tonic GABA current, when the concentration of extracellular GABA was raised by adding GABA to the bath along with the GAT-1 blocker NNC711, was less than that observed in DGGCs. Previously, Milenkovic et al. (2013) found ubiquitous expression GABA_A_R δ-subunits, high α_1_ and low α_4_ GABA_A_R subunit expression and strong co-localization of α_1_-, β_2_-, and δ-subunits in GCL PV^+^-INs. This data strongly suggested that these interneurons express α_1_/δ eGABA_A_R as has been described previously for interneurons of the ML of the DG (Glykys et al., 2007). In the *Xenopus* oocyte expression system, it has been shown that α_1_β_3_δ GABA_A_R have a markedly lower affinity for GABA [EC_50_: 8.7 μM (Karim et al., 2012); EC_50_: 8.5 μM (Kaur et al., 2009)] than α_4_β_2_δ containing GABA_A_R [EC_50_: 1 μM (Karim et al., 2012); EC_50_: 0.41 μM (Wongsamitkul et al., 2017)] but that both of these δ-subunit containing GABA_A_R display properties of constitutive activity (Karim et al., 2012). This latter finding has been confirmed in rat DGGC where tonic currents at resting extracellular GABA levels appear to be largely mediated by δ-subunit containing GABA_A_R displaying GABA-independent channel openings (Wlodarczyk et al., 2013). Moreover, in mammalian expression systems, when GABA is used as the agonist, α_1_β_3_δ and α_4_β_2_δ GABA_A_R have been shown to have similar single channel conductances (Fisher and MacDonald, 1997; Keramidas and Harrison, 2008) and in DGGC expressing α_4_β_2_δ receptors GABA-independent single channel currents are equivalent size to those in the presence of GABA (Wlodarczyk et al., 2013). Thus, based on our finding of similar normalized basal tonic currents in DGGCs (1.4 ± 0.4 pA/pF) and PV^+^-INs (1.2 ± 0.1 pA/pF) and the previously reported similarities in α_4_/δ and α_1_/δ receptor single channel conductances, we suggest that these cells express roughly similar densities of α_4_ and α_1_ containing eGABA_A_R, respectively. We propose that at resting extracellular GABA levels, tonic currents in both DGGCs and PV^+^-INs are mediated by constitutive activity relying largely on GABA independent channel openings. This conclusion stems from the fact that the ambient level of extracellular GABA in the DG has been shown to be sufficiently low to indicate that the majority of tonic inhibition in DGGCs, which is mediated by higher affinity α_4_β_2_δ, is via GABA-independent channel openings (Wlodarczyk et al., 2013). Thus, at low basal ambient GABA levels, where a reasonably large tonic current is observed in PV^+^-INs, it is unlikely that lower affinity α_1_β_X_δ eGABA_A_R would be strongly activated by the neurotransmitter while higher affinity α_4_β_2_δ receptors in DGGC are not. However, as the extracellular GABA concentration is elevated, the differential expression of eGABA_A_R with substantially different GABA affinities (α_4_β_2_δ ≫ α_1_β_X_δ) between the two type of cells means that tonic currents are more strongly enhanced in DGGCs compared to PV^+^-INs (i.e., the concentration response curve for GABA is significantly left-shifted in DGGCs vs PV^+^-INs). It is unclear precisely what the physiologic function of this difference in eGABA_A_R composition is. One possibility is that lower sensitivity to GABA in fast-spiking PV^+^-INs might permit these cells to elevate the level of extracellular GABA in the GCL, via perisomatic release of GABA, to a point where it can effectively enhance tonic inhibition to DGGCs and reduce their firing without producing substantial tonic inhibitory feedback onto themselves or other PV^+^-INs that are in close physical proximity in the GCL. This could allow PV^+^-INs to respond to strong excitation by providing substantial feedforward inhibition to DGGCs. On the other hand, PV^+^-INs in the GCL have dense axonal projections in the GCL itself and are typically perisoma-inhibiting cells (Hu et al., 2014). Whereas, the expression of α_4_- and δ-subunits, and therefore α_4_β_2_δ eGABA_A_R, in DGGCs is greatest in their dendrites in the ML, the strongest expression of α_1_- and δ-subunits in GCL PV^+^-INs is at the soma (Milenkovic et al., 2013). Thus, by expressing lower affinity somatic eGABA_A_R, when PV^+^-INs fire strongly, as happens for example during γ-oscillations, they can provide robust temporally precise synaptic inhibition to the soma of nearby DGGCs (Strüber et al., 2015, 2017), without the resulting GABA spill-over impacting substantially on their own firing allowing them to maintain precise control of the timing of DGGC firing. Nonetheless, the presence of eGABA_A_R, albeit lower affinity ones, in GCL PV^+^-INs would still provide a valuable auto-inhibitory backstop to regulate their own output should the extracellular GABA concentration become sufficiently elevated.

Our new findings reveal that tonic inhibition in GCL PV^+^-INs, unlike DGGCs, is sensitive to the α_1_-selective drug zolpidem. Thus, it is highly likely that at least a fraction of the tonic inhibition in PV^+^-INs, unlike DGGCs, is mediated by receptors containing α_1_-subunits, possibly in arrangements containing δ-subunits or as αβ pentamers. Although it is conventionally thought that zolpidem binding requires the presence of γ-subunits that are not commonly found in eGABA_A_Rs, recent evidence has demonstrated a novel binding site for zolpidem at the interface between two α_1_-subunits (Che Has et al., 2016), further supporting the existence of α_1_β_X_δ eGABA_A_R. Thus, PV^+^-INs may express distinct populations of eGABA_A_R with unique pharmacological properties that would allow them to be targets for new subunit-selective GABA modulating drugs. These receptors might represent novel targets for treatment of neurodevelopmental and neuropsychiatric disorders where PV^+^-INs have long been implicated in disease (Lewis et al., 2012; Nakazawa et al., 2012).

## Synthesis

Reviewing Editor: Alfonso Represa, INSERM Decisions are customarily a result of the Reviewing Editor and the peer reviewers coming together and discussing their recommendations until a consensus is reached. When revisions are invited, a fact-based synthesis statement explaining their decision and outlining what is needed to prepare a revision will be listed below. The following reviewer(s) agreed to reveal their identity: Zoltan Nusser.

This MS examines the effect of haploinsufficiency for CYFIP1 on GABAA receptor ∂-subunit expression and phasic and tonic inhibition in the gyrus dentatus. The authors recorded spontaneous IPSCs an tonic current from granule cells and PV+ interneurons in acute brain slices from control and CYFIP1+/- mice and found no significant difference between the two genotypes.

These experiments were designed, performed and analyzed well, without any apparent shortcomings. Overall, the data indicate that the mouse model for the Cyfip1 haploinsufficiency, mimicking human 15q11.2 microdeletion syndrome, doesn't show a deficit in tonic or phasic GABAergic transmission. In addition, the authors demonstrated that the a1 subunit-selective drug Zolpidem increases tonic inhibition in PV+ INs, but not in granule cells. The interpretation of these experiments is wide open.

In the introduction, the authors elegantly describe the devastating effects of the loss of FMRP on behavior and cognition. It was surprising to read that the authors do not provide any information about the effects of CYFIP1 deletion in the behavior of the mice.

In the introduction, the role of FMRP in GABAergic transmission is described. However, the literature supporting the FMRP-CYFIP1 regulatory complex is not well stated. In particular out of the ~800 mRNA regulated by with FMRP, only a portion of them is present in the FMRP-Cyfip1 complex (Napoli et al, 2008). Therefore, not all of the FMRP targets are found dysregulated in the Cyfip1 haploinsufficiency mouse model. Additionally, the authors should provide a more extensive description of the similarity and specific features of the FXS and 15q11.2 deletion syndrome to possibly explain the absence of altered GABAergic transmission in the Cyfip1 haploinsufficiency mouse model. Finally, the introduction lacks details on the role of Cyfip1 and its implication in the 15q11.2 deletion syndrome. This should be added in the introduction.

A recent paper published from Kittler's laboratory (https://doi.org/10.1101/303446) demonstrates that Cyfip1 loss leads to: increased inhibitory postsynaptic clustering, enhanced expression of GABAA receptor β-subunits, and an increased in mIPSC amplitude in CA1 hippocampal cells. These data need to be considered and discussed before proposing as conclusion on the role of Cyfip1 in GABAergic transmission.

In figure 2 the authors recorded the THIP evoked currents in DGGC of WT and Cyfip1 haploinsufficiency mice. This doesn't fully explain the kinetics of GABAA receptors in response to THIP treatment. A dose-response curve should be added in order to measure the specificity of the GABAA receptor subunit response. Moreover, the authors should explain better how panels C-F were generated. It is not clear which transformation has been applied to the data from panels A and B in order to generate panels C-F. Moreover, from panels A and B (right plot) the pA values for THIP 3µM and THIP 10µM looks different when comparing WT and Cyfip1 haploinsufficiency traces.

The authors claim that PV+ INs have substantial tonic inhibition and that the extrasynaptic GABAARs underlying this tonic inhibition contain the delta subunit as revealed by its sensitivity to THIP (line 528). This is a widely used misconception in the field. The fact that the delta selective agonist increases tonic current does not tell anything about the tonic current under control condition. It could originate from synaptic or extrasynaptic receptors of unknown subunit composition. How could the authors (or anyone) exclude the possibility that synaptic a2b3g2 receptors provide the tonic inhibition in control conditions? Just because they can recruit extrasynaptic delta containing ones with THIP, it tells nothing about the original current.

A very similar misconception is found when interpreting the effect of zolpidem. The fact that zolpidem increased the tonic current, the authors concluded that they must act on receptors that produce the tonic current. Why? The tonic current could be produced by receptors X at Y location, and zolpidem could increase the affinity of receptors A at B location? Why is this possibility less likely? In fact, zolpidem and BZ binding requires the g2 subunit, therefore it is more likely that synaptic a1b2g2 channels underlie the increase in tonic current following zolpidem application.

The results of the experiments with the GABA uptake inhibitor and 5 μM GABA is also misinterpreted. The authors take these results as evidence for a higher affinity of GABAA receptors. Why is it more likely than more receptors? Why is it more likely than activating receptors with higher efficacy? It is well known that the delta containing receptors have high affinity, but very low efficacy, but other receptors with lower affinity, but much higher efficacy could produce a larger current.

Examining the total mRNA in the hippocampus for the delta and a4 subunits might be logical, because the CA1 and CA3 regions have very little expression. However, the use of synaptosomal preparations would provide more accurate data. Analyzing the a1 subunit mRNA in the whole hippocampus seems to be a very crude approach. It is well known that all principal cells express the a1 subunit and they outnumber the PV+ INs, therefore a potential change in the INs could remain unnoticed.

In the first paragraph in page 19, the authors describe very briefly experiments in which they examined protein levels. Why did the authors concentrate on mRNA instead of the protein for the rest of the MS? The level of protein is clearly functionally more relevant than that of mRNA!

For the quantification of the Western blots, authors must clarify how data were normalized. In addition, the bands for the delta subunit are hardly visible in fig 5D. How much was the background and was the background subtracted from the signal? If the authors prepared membrane fractions from the CA1 area, what signal would they get for the delta?

The authors should clarify for each figure (in the figure legend) how many cells -and from how many mice - were used for each experiment together and the age of the mice used.

The Y scales in figure 6 panel D-F do not make sense! Please use a scale that covers the range of the data and not one that is 10 times larger!

Text in line 320 is mixed up!
